# Salivary Gland Dysfunction, Protein Glycooxidation and Nitrosative Stress in Children with Chronic Kidney Disease

**DOI:** 10.3390/jcm9051285

**Published:** 2020-04-29

**Authors:** Mateusz Maciejczyk, Julita Szulimowska, Katarzyna Taranta-Janusz, Anna Wasilewska, Anna Zalewska

**Affiliations:** 1 Department of Hygiene, Epidemiology and Ergonomics, Medical University of Bialystok, 2c Mickiewicza Street, 15-233 Bialystok, Poland; 2 Department of Pedodontics, Medical University of Bialystok, 24a M. Sklodowskiej-Curie Street, 15-274 Bialystok, Poland; szulimowska.julita@gmail.com; 3 Department of Pediatrics and Nephrology, Medical University of Bialystok, 24a M. Sklodowskiej-Curie Street, 15-274 Bialystok, Poland; katarzyna.taranta@wp.pl (K.T.-J.); annwasil@interia.pl (A.W.); 4 Experimental Dentistry Laboratory, Medical University of Bialystok, 24a M. Sklodowskiej-Curie Street, 15-274 Bialystok, Poland or azalewska426@gmail.com

**Keywords:** chronic kidney disease, salivary gland dysfunction, salivary biomarkers, oxidative stress, nitrosative stress

## Abstract

This study is the first to evaluate protein glycooxidation products, lipid oxidative damage and nitrosative stress in non-stimulated (NWS) and stimulated whole saliva (SWS) of children with chronic kidney disease (CKD) divided into two subgroups: normal salivary secretion (*n* = 18) and hyposalivation (NWS flow < 0.2 mL min^−1^; *n* = 12). Hyposalivation was observed in all patients with severe renal failure (4–5 stage CKD), while saliva secretion > 0.2 mL/min in children with mild-moderate CKD (1–3 stage) and controls. Salivary amylase activity and total protein content were significantly lower in CKD children with hyposalivation compared to CKD patients with normal saliva secretion and control group. The fluorescence of protein glycooxidation products (kynurenine, N-formylkynurenine, advanced glycation end products), the content of oxidative damage to lipids (4-hydroxynonneal, 8-isoprostanes) and nitrosative stress (peroxynitrite, nitrotyrosine) were significantly higher in NWS, SWS, and plasma of CKD children with hyposalivation compared to patients with normal salivary secretion and healthy controls. In CKD group, salivary oxidation products correlated negatively with salivary flow rate, α-amylase activity and total protein content; however, salivary oxidation products do not reflect their plasma level. In conclusion, children with CKD suffer from salivary gland dysfunction. Oxidation of salivary proteins and lipids increases with CKD progression and deterioration of salivary gland function.

## 1. Introduction

Chronic kidney disease (CKD) is a multi-symptomatic syndrome resulting from a reduction in the number of active nephrons. The diagnosis of CKD is based on anatomical and/or functional renal abnormalities as well as glomerular filtration rate (GFR) below 60 mL/min/1.73 m^2^ [1]. Although the prevalence of CKD in children is much lower than in adults, the disease is a significant clinical problem in the child population. Indeed, mortality in CKD children remains high and is about 30 times higher than the expected mortality at any given age [2]. The most common causes of CKD in children are urological defects, glomerulopathies, congenital nephropathies, and kidney dysplasia [2,3]. Their effect is the reduction of active nephrons, leading to intraglomerular hypertension in the remaining nephrons and their hypertrophy. This also leads to proteinuria, progressive hardening of the glomeruli as well as fibrosis of the renal interstitial tissue [1,4]. However, CKD complications can affect just about every organ [1]. These include cardiovascular disease (hypertrophy of the left ventricle, coronary heart disease), respiratory system (pulmonary edema, “uremic lung”), endocrine disorders (glucose intolerance, dyslipidemia), hematological (normochromic anemia, hemorrhagic diathesis) or mineral and bone disorders (vitamin D deficiency, hypoparathyroidism) [1,4]. In the CKD pathogenesis, the key role of oxidative stress has recently been stressed [5,6,7].

The increased production of free radicals in CKD leads to oxidative stress which initiates oxidative damage to proteins and lipids. This increases the accumulation of oxidized proteins in the kidney parenchyma and leads to a progressive impairment of its function [5,6,7]. It has been proven that the advanced oxidation protein products (AOPP) and advanced glycation end products (AGE) intensify the RAAS (renin–angiotensin–aldosterone system) activation, increase the expression of NF-κB (nuclear factor-κB) pathway and impair nitric oxide (NO) production [8,9]. The oxidation protein products increase synthesis of collagen and fibronectin in the mesangial cells, activate the NADPH oxidase (NOX) through the protein kinase C dependent pathway, enhance the activity of caspase-3, the expression of the p58 protein and Bax. Therefore, the protein oxidation products play a critical role in proteinuria and thickening of the renal glomeruli progression, decreasing the number of podocytes through apoptosis [10,11,12,13]. Moreover, as a result of peroxidation of kidney lipids, the activity of membrane enzymes and transporting proteins is inhibited, which disturbs the integrity of cell membranes [5,6,7]. Nevertheless, it is suggested that the accumulation of oxidized proteins and lipids may also disrupt other organs [5,6,7].

A number of systemic diseases affect the function of salivary glands. Reduced saliva production, disturbances of protein secretion into saliva as well as xerostomia (subjective dryness of oral mucosa) were observed in patients with diabetes, obesity, hypertension, psoriasis, and rheumatoid arthritis [14,15,16,17,18]. It is suggested that oxidative stress may play a key role in the pathogenesis of salivary hypofunction. In fact, the oral cavity is the only place in the body exposed to so many environmental factors such as food, stimulants (alcohol, tobacco smoke), air pollution, medicines, or dental materials [19]. Although all of them can generate oxygen free radicals, patients with systemic diseases are particularly predisposed to salivary oxidative stress [14,15,16,17]. Indeed, in a situation of reduced antioxidant capacity, systemic oxidative stress can affect the oxidative-reductive balance of the oral cavity. Products of protein/lipid oxidation can aggregate and accumulate in the salivary glands leading to damage of secretory cells. Protein oxidation products can also increase reactive oxygen species (ROS) formation (by activating NOX and NF-κB signaling), which, on a positive feedback, enhances local oxidative stress [20,21].

In our earlier studies we have shown that oxidative stress in CKD children affects not only the kidneys but also the oral cavity [3,22,23]. Indeed, we have shown disturbances of the enzymatic and non-enzymatic antioxidant barrier and increased oxidative damage to salivary proteins [3]. Moreover, salivary FRAP (ferric ion reducing antioxidant power) with high sensitivity (100%) and specificity (100%) differentiates children with mildly to moderately decreased kidney function from those with severe renal impairment [22]. Additionally, CKD patients are much more likely to develop oral diseases such as dental caries, candidiasis or tooth erosion [24]. However, still little is known about salivary gland function in children with CKD. We suppose that as in other oxidative stress-related diseases, CKD causes a decrease in saliva production and disturbances of protein secretion into saliva [20,25,26,27]. This may be due to the accumulation of protein oxidation products in the salivary glands, which damage their parenchyma and lead to hyposalivation. As in obesity, insulin resistance or psoriasis, salivary gland hypofunction may also result from the impairment of NO bioavailability and the damaging effect of nitrosative stress mediators (especially peroxynitrite) [20,25,26]. Therefore, our study is the first to evaluate salivary glycooxidation products, oxidative damage to lipids and nitrosative stress biomarkers in CKD children with normal and decreased saliva secretion. In addition to the non-stimulated and stimulated salivary flow, we also assessed other indicators of salivary gland function, such as salivary amylase activity and total protein content. An important part of our study is also the assessment of salivary-blood correlation of the analyzed redox biomarkers.

## 2. Material and Methods

### 2.1. Ethical Issues

The study was approved by the Local Bioethics Committee at the Medical University of Bialystok (permission number R-I-002/43/2018). All patients and/or their legal guardians have been acquainted with the research project and gave written consent to participate in the experiment.

### 2.2. Patients

The study included 30 children with CKD treated in the Department of Pediatrics and Nephrology of the Medical University of Bialystok, Poland. Patients were divided into two subgroups based on the rate of non-stimulated salivary flow (NWS): normal salivary secretion (normal salivation, CKD NS) and reduced salivary secretion (hyposalivation; CKD HS). Hyposalivation was defined as NWS flow below 0.2 mL/min [16,26,28].

CKD was defined according to the Kidney Disease Improving Global Outcomes (KDIGO) criteria based on different eGFR distribution: Stage 1: >90 mL/min/1.73 m^2^; Stage 2: 60–89 mL/min/1.73 m^2^; Stage 3: 30–59 mL/min/1.73 m^2^; Stage 4: 15–29 mL/min/1.73 m^2^; and Stage 5: <15 mL/min/1.73 m^2^ [1]. The estimated glomerular filtration rate (eGFR) was calculated using the updated Schwartz formula-eGFR (mL/min/1.73 m^2^) = 0.413 × (height in cm/serum creatinine (Cr)) [29]. Upon the diagnosis of CKD, all patients were on a renal diet that was low in sodium and/or phosphorous and/or protein depending on patients’ condition and CKD stage [30]. Office blood pressure (BP) was measured by means of either the manual auscultatory technique or an automated oscillometric device after the subject had rested for 5 min in a sitting position. The average values of the second and third measurements of systolic and diastolic BP were used. Hypertension was defined when the average value of the systolic and/or diastolic BP were ≥95th percentile for age, gender, and height [31]. 

The causes of CKD were urological defects (33.3%), glomerulopathies (33.3%), congenital nephropathies (13.3%), kidney dysplasia (13.3%), and undetermined etiology (6.8%).

The control group consisted of 30 healthy children attending the Specialist Dental Clinic of the Medical University of Bialystok, Poland for regular check-ups. The control was matched by age and gender to the study group. All patients in the control group had an NWS flow > 0.2 mL/min.

The exclusion criterion in the study and control groups was the occurrence of general diseases: metabolic (insulin resistance, type 1 and 2 diabetes), autoimmune (thyroiditis, systemic sclerosis, arthritis, lupus erythematosus, Crohn’s disease, ulcerative colitis), infectious, gastrointestinal and pulmonary diseases. Patients taking antibiotics, non-steroidal anti-inflammatory drugs (NSAIDs), glucocorticosteroids, vitamins and dietary supplements for at least 1 months before saliva collection were excluded from the study, similarly to children with acute inflammatory states. Subjects with poor oral hygiene (Approximal Plaque Index, API > 20) and gingivitis (Sulcus Bleeding Index, SBI > 0.5; Gingival Index, GI > 0.5) were also excluded from the experiment (see: dental examination).

Since pharmacotherapy significantly affects saliva secretion [16,27], patients with CKD taking 5 and more drugs were eliminated from the study. 

Detailed characteristics of the patients and the control group are presented in Table 1.

### 2.3. Saliva Collection

The research material was non-stimulated (NWS) and stimulated (SWS) whole saliva collected by the spitting method. In order to eliminate the influence of physical exercise and daily rhythm on saliva secretion, samples were taken from subjects who were not physically active for the last 24 h, after an all-night rest, always between 7 a.m. and 9 a.m. Subjects did not consume any meals or drinks (other than water), and refrained from performing any oral hygiene procedures at least 2 h before saliva collection. Additionally, children did not take any medications for at least 8 h prior to saliva collection [16,28].

The subjects were instructed to rinse their mouth two times with distilled water and to spit saliva into a sterile Falcon tube placed in an ice container. Saliva collection was done by the patient when sitting with the head down (with minimized facial and lip movements), after at least a 5-min adaptation, always in the same child-friendly room. The time of NWS collection was 15 min. Then, saliva was stimulated by dropping 10 µl of citric acid (2%, w/v) solution on the tip of the tongue every 30 s [16,17,26,28]. The time of SWS collection was 5 min [16,28].

Immediately after collection, the volume of saliva was measured with a pipette set to 100 µL. The salivary flow rate was calculated by dividing the volume of saliva by the time necessary for its secretion (mL min^−1^). The pH of saliva was also analyzed using Seven Multi pH meter (Mettler Toledo, Greifensee, Switzerland).

After measuring the salivary pH, the samples were immediately centrifuged (3000× *g*, 4 °C, 20 min) and the supernatant was preserved for further analysis [32]. To protect against sample oxidation, butylated hydroxytoluene (BHT, Sigma-Aldrich, Nümbrecht, Germany) was added (10 μL 0.5 M BHT in acetonitrile (ACN)/1 mL NWS/SWS) [32]. The samples were portioned into 200 μL aliquots and frozen at −80 °C. Frozen samples were stored for no more than six months. 

In order to identify samples contaminated with blood, the concentration of transferrin in saliva was assessed (Human Transferrin ELISA Kit; Abcam; Cambridge, UK). However, no blood contamination was confirmed in any of the samples.

The activity of salivary amylase (EC 3.2.1.1) was assessed for the evaluation of salivary gland function [33,34]. A spectrophotometric method with 3,5-dinitrosalicylic acid (DNS) was used and absorbance was measured at 540 nm. 

### 2.4. Dental Examination

A clinical examination in artificial lighting (10,000 lux) was also performed. According to the World Health Organization criteria [35], a mirror, an explorer and a periodontal probe were used. The incidence of caries was assessed using DMFT index (decay, missing, filled teeth). DMFT is the sum of teeth with caries (D), teeth extracted because of caries (M), and teeth filled because of caries (F). DMFT for deciduous teeth (dmft) was also evaluated. API (approximal plaque index) was used to assess the status of oral hygiene and determines the percentage of tooth surface with plaque. GI (Gingival Index) and SBI (Sulcus Bleeding Index) were used to assess the condition of gums. GI described qualitative changes in the gingiva, while SBI showed the intensity of bleeding from the gingival sulcus after probing [35].

Clinical dental examinations were performed by the same experienced pedodontist (J. S.). In 10 children, the inter-rater agreements between the examiner and another experienced pedodontist (A. Z.) were assessed. The reliability for all dental indices was >0.97.

### 2.5. Blood Collection

Whole blood was collected after an all-night rest, always between 6 and 8 a.m. We used S-Monovette^®^ K3 EDTA blood collection system (Sarstedt, Nümbrecht, Germany). Samples were immediately centrifuged (1500× *g*; 4 ℃, 10 min) [32] and the top layer (plasma) was preserved for further analyses. Similarly to NWS and SWS, BHT (10 μL 0.5 M BHT/1 mL plasma) was added to samples that were then frozen at −80 °C [32].

### 2.6. Total Protein Assay

The total protein content was determined colorimetrically using the bicinchoninic acid (BCA) method (Thermo Scientific PIERCE BCA Protein Assay (Rockford, IL, USA)). Bovine serum albumin (BSA) was used as a standard.

### 2.7. Redox Assays

All reagents were purchased from Sigma-Aldrich (Nümbrecht, Germany and/or Saint Louis, MO, USA). The absorbance/fluorescence was measured using Infinite M200 PRO Multimode Microplate Reader Tecan. The results were standardized to 1 mg of total protein. All determinations were performed in duplicate samples.

### 2.8. Protein Glycooxidation Products

The content of dityrosine, kynurenine, N-formylkynurenine and tryptophan was assessed fluorimetrically. The characteristic fluorescence at 330/415, 365/480, 325/434, and 295/340 nm, respectively, was measured [36,37]. Immediately before determination, saliva and plasma samples were diluted in 0.1 M H_2_SO_4_ (1:5, v/v) [32]. The results were normalized to fluorescence of 0.1 mg/mL quinine sulfate in 0.1 M H_2_SO_4_ and expressed in arbitrary fluorescence units (AFU)/mg protein.

The content of advanced glycation end products (AGE) was assessed fluorimetrically. The characteristic fluorescence of pentosidine, pyraline, carboxymethyl lysine (CML), and furyl-furanyl-imidazole (FFI) was measured at 350/440 nm [38]. Immediately before determination, saliva and plasma samples were diluted in 0.1 M H_2_SO_4_ (1:5, v/v) [32]. The results were expressed in arbitrary fluorescence units (AFU)/mg protein.

### 2.9. Oxidative Stress Products

The total thiols concentration was measured colorimetrically using the Ellman’s reagent (5,5-dithio-bis-(2-nitrobenzoic acid)) [39]. The absorbance was measured at 412 nm and total thiols concentration was expressed in μmol/mg protein.

4-hydroxynonneal protein adducts (4-HNE) and 8-isoprostanes (8-isop) concentration was measured using ELISA kits (Cell Biolabs, Inc. San Diego, CA, USA; Cayman Chemicals, Ann Arbor, MI, USA, respectively), following the manufacturer’s instructions. The results were expressed in nmol/mg protein and pmol/mg protein, respectively.

### 2.10. Nitrosative Stress Products

Nitric oxide (NO) concentration was measured colorimetrically using sulfanilamide and NEDA·2 HCl (N-(1-naphthyl)-ethylenediamine dihydrochloride). Nitrate was converted to nitrite using nitrate reductase and total NO was measured [40,41]. The absorbance was measured at 490 nm and NO concentration was expressed in μmol/mg protein.

S-nitrosothiols concentration was measured colorimetrically based on the reaction of the Griess reagent with Cu^2+^ ions [41,42]. The absorbance was measured at 490 nm and S-nitrosothiols concentration was expressed in nmol/mg protein.

Peroxynitrite concentration was measured colorimetrically based on peroxynitrite-mediated nitration resulting in the formation of nitrophenol [43]. The absorbance was measured at 320 nm and peroxynitrite concentration was expressed in nmol/mg protein.

Nitrotyrosine concentration was measured colorimetrically by the ELISA method, using a commercial diagnostic kit (Immundiagnostik AG; Bensheim, Germany). Nitrotyrosine concentration was expressed in pmol/mg protein.

### 2.11. Statistical Analysis

Statistical analysis was performed using GraphPad Prism 8 for Mac (GraphPad Software, La Jolla, USA). The Shapiro–Wilk test was used to determine the normality of distribution while one-way ANOVA and Tukey’s multiple comparisons test were used to compare the tested groups. The value of *p* < 0.05 was considered statistically significant. Multiplicity adjusted p value vas also calculated. The results were presented as mean ± SD. The correlation of the obtained results was measured using the Pearson correlation coefficient. The number of patients was set a priori based on the previous clinical study. Online sample size calculator (ClinCalc) was used and 0.9 was assumed as the test power.

## 3. Results

### 3.1. Clinical Characteristics

Clinical characteristics of the subjects are presented in Table 1.

Interestingly, hyposalivation was observed in all patients with severe renal failure (4–5 stage CKD), while saliva secretion >0.2 mL/min in children with mild-moderate CKD (1–3 stage) and controls.

### 3.2. Salivary Gland Function and Salivary pH

**Figure 1:**
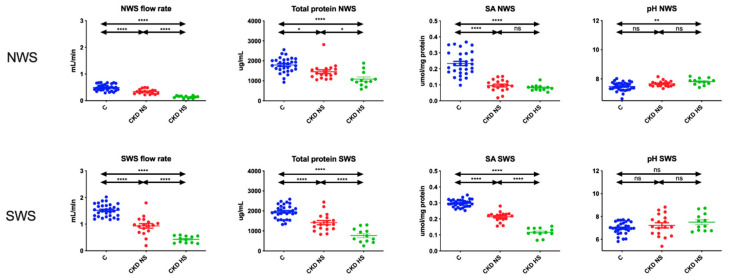
Salivary gland function and salivary pH of children with chronic kidney disease (CKD) and healthy controls. C—Healthy controls; CKD NS—CKD patients with normal salivary secretion; CKD HS—CKD patients with reduced salivary secretion; NWS—Non-stimulated whole saliva; SA—Salivary amylase; SWS—Stimulated whole saliva. Differences statistically significant at: * *p* < 0.05, ** *p* < 0.005, **** *p* < 0.0001.

The non-stimulated and stimulated salivary secretion was significantly lower in CKD children with hyposalivation compared to patients with normal salivary secretion and control group. Similarly, total protein content and salivary amylase activity were significantly lower in NWS and SWS of CKD children with hyposalivation as compared to other groups. The pH of non-stimulated saliva was significantly higher in CKD children with decreased salivary secretion compared to controls (Figure 1).

### 3.3. Dental Examination

Oral hygiene (DMFT, dmft, API) and periodontal condition (GI, SBI) did not differ significantly between groups (Table 2). The children had all permanent teeth completely erupted (up to the seventh tooth). There was no active eruption of eighth teeth in any child.

### 3.4. Glycooxidation Products

**Figure 2:**
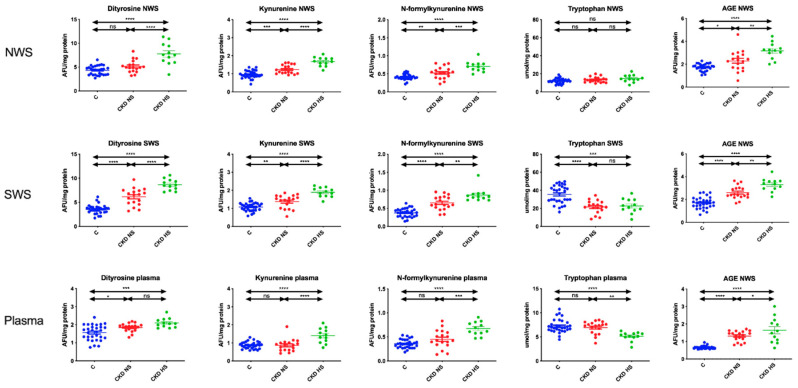
Glycooxidation products in children with chronic kidney disease (CKD) and healthy controls. AGE—Advanced glycation end products; C—Healthy controls; CKD NS—CKD patients with normal salivary secretion; CKD HS—CKD patients with reduced salivary secretion; NWS—Non-stimulated whole saliva; SWS—Stimulated whole saliva. Differences statistically significant at: * *p* < 0.05, ** *p* < 0.005, *** *p* < 0.0005, **** *p* < 0.0001.

Generally, the fluorescence of glycooxidation products (dityrosine, kynurenine, N-formylkynurenine and AGE) was significantly higher in NWS, SWS and plasma of CKD children with hyposalivation compared to patients with normal salivary secretion and control group. Tryptophan fluorescence was significantly lower in stimulated saliva and plasma of patients with CKD (both groups) as compared to controls (Figure 2).

### 3.5. Oxidative Stress Products

**Figure 3:**
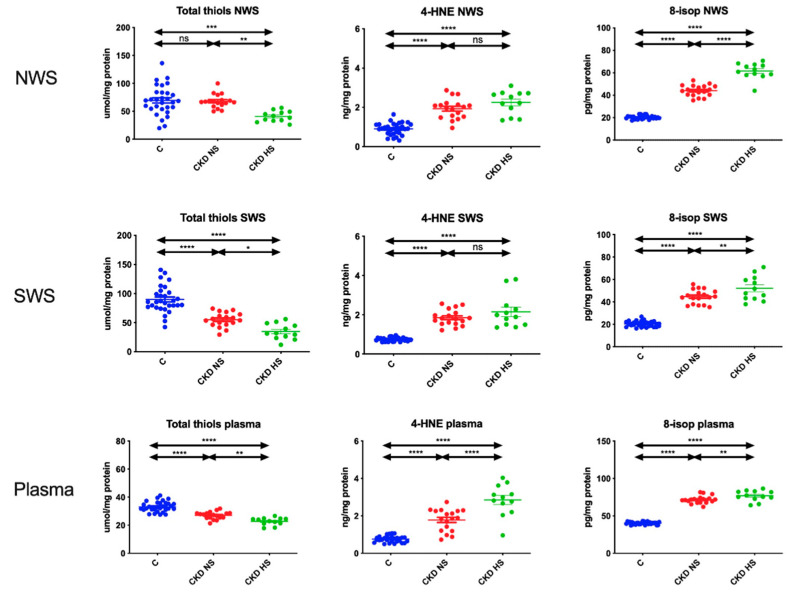
Oxidative damage to proteins and lipids in children with chronic kidney disease (CKD) and healthy controls. 4-HNE—4-hydroxynoneal protein adducts; 8-isop—8-isoprostanes; C—Healthy controls; CKD NS—CKD patients with normal salivary secretion; CKD HS—CKD patients with reduced salivary secretion; NWS—Non-stimulated whole saliva; SWS—Stimulated whole saliva. Differences statistically significant at: * *p* < 0.05, ** *p* < 0.005, *** *p* < 0.0005, **** *p* < 0.0001.

Oxidative damage to proteins (total thiols) and lipids (4-HNE and 8-isop) was significantly higher in NWS, SWS and plasma of children with chronic kidney disease and hyposalivation compared to patients with normal salivary secretion and control group (Figure 3).

### 3.6. Nitrosative Stress Products

**Figure 4:**
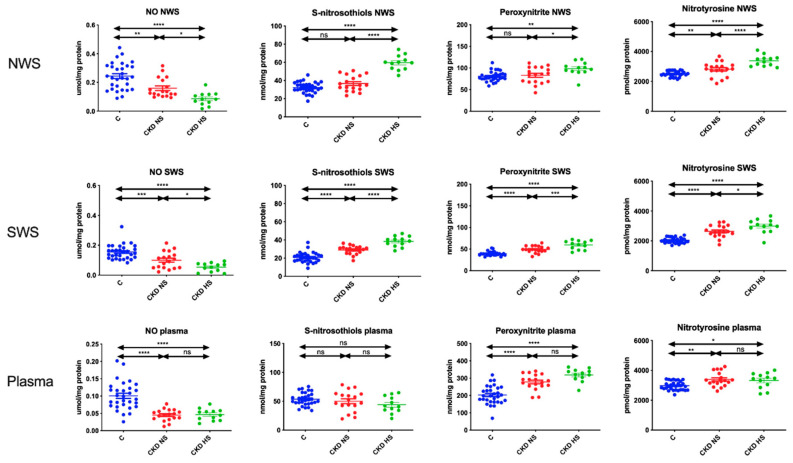
Nitrosative stress in children with chronic kidney disease (CKD) and healthy controls. CKD NS—CKD patients with normal salivary secretion; CKD HS—CKD patients with reduced salivary secretion; NO—nitric oxide; NWS—non-stimulated whole saliva; SWS—stimulated whole saliva. Differences statistically significant at: * *p* < 0.05, ** *p* < 0.005, **** *p* < 0.0001.

NO concentration was significantly lower in NWS, SWS, and plasma in CKD children with hyposalivation compared to other groups. The concentration of S-nitrosothiols was significantly higher in NWS and SWS of children with chronic kidney disease and hyposalivation compared to CKD patients with normal salivary secretion and healthy controls. However, it did not differ significantly in plasma. The content of peroxynitrite and nitrotyrosine was significantly higher in NWS, SWS and plasma of CKD children with hyposalivation in comparison with the other groups (Figure 4).

### 3.7. Correlations

In CKD children the concentration of redox biomarkers in NWS and SWS correlated negatively with eGFR and positively with serum creatinine and urea (except for total thiols and tryptophan). However, no correlation with renal function parameters was generally observed in healthy subjects (Table 3).

In children with CKD, the concentration of protein and lipid oxidation products correlates negatively with salivary flow rate, salivary amylase activity and total protein content. Only salivary tryptophan and thiol groups correlated positively with salivary glands activity (Table 4). In NWS, glycooxidation products (except tryptophan), 4-HNE, 8-isop and peroxynitrite correlated positively with salivary pH. However, there was no relationship between the assessed biomarkers in saliva and plasma (except for kynurenine) (Table 5).

In the control group, dityrosine, kynurenine, tryptophan, AGE, and 4-HNE in non-stimulated saliva correlates positively with their plasma levels (Table 5). However, no relationships between cellular oxidation products and salivary gland function were demonstrated (except for AGE in SWS) (Table 4).

## 4. Discussion

This study is the first to evaluate protein glycooxidation products, lipid oxidative damage and nitrosative stress in non-stimulated and stimulated saliva and plasma in children with chronic kidney disease. We have shown that in CKD there is a dysfunction of salivary glands, which intensifies with the oxidation of salivary proteins/lipids and nitrosative damage. Interestingly, in children with CKD, salivary oxidation products did not correlate with their plasma content. Therefore, disturbances in salivary redox homeostasis may occur independently of alterations at the central level (plasma).

In the course of CKD, pathological changes of oral mucosa, susceptibility to fungal infections and olfactory and taste disorders were observed [44]. Therefore, it is not surprising that non-stimulated and stimulated saliva secretion was significantly lower in all children with CKD in comparison to controls. However, hyposalivation (NWS flow < 0.2 mL/min) was observed only in patients with severe renal failure (4–5 stage CKD). This indicates the progression of salivary hypofunction according to the CKD severity. This may also explain the increased incidence of dental caries and periodontal disease in children with advanced stages of CKD [44]. However, total protein content and salivary amylase activity were also significantly lower in CKD children with hyposalivation compared to other subjects (CKD children with normal salivary secretion and healthy controls). Indeed, it should be recalled that α-amylase is not only involved in the degradation of food polysaccharides [33]. This enzyme is synthesized in acinar cells (i.e., major secretory cells) of the salivary glands, where it is stored in granules before secretion. Secretory granules are transported to the apical membrane, fuse with the membrane and secrete their contents into the secretory ducts by exocytosis. Therefore, α-amylase can be an indicator of protein secretion through exocytosis [33,45]. Many studies have also shown a relationship between the decrease in α-amylase activity and impairment of secretory function of salivary glands [17,33]. Consequently, CKD is not only associated with reduced saliva secretion, but also with impaired protein secretion into saliva (Figure 1).

Salivary oxidative/nitrosative stress was significantly higher in children with CKD and hyposalivation (4–5 stage CKD) compared to patients with normal salivary secretion (1–3 stage CKD). Therefore, both salivary glycooxidation and lipoperoxidation intensify with the progression of CKD. Interestingly, salivary oxidation products correlate negatively with salivary flow rate, α-amylase activity and total protein content.

As a result of protein oxidation, many structural and functional changes occur. Indeed, the amino acids are modified, the protein chain is fragmented and cross-linkages between the amino acids are formed. Interestingly, thiol groups (-SH) are the first to be oxidized [46]. Thus, it is no surprising that the salivary thiol levels in CKD have decreased. However, ROS also react with side chains of amino acids (e.g., tyrosine, lysine, arginine, or threonine). In our study we observed a quenching of tryptophan fluorescence (probably due to enhanced oxidation of salivary albumin [47]) as well as an increase in the fluorescence of protein glycooxidation products. Interestingly, the fluorescence of glycooxidation products was significantly higher in both NWS and SWS of CKD children with hyposalivation (↑dityrosine, ↑kynurenine, ↑N-formylkynurenine, and ↑AGE) compared to patients with normal salivary secretion. Under inflammatory conditions, there is an increased production of reactive chlorates that combine with tyrosine or kynurenine. The resulting aggregates tend to accumulate in the tissues [46]. AGE, products of non-enzymatic glycation of proteins, can react with a specific receptor (RAGE, receptor for advanced glycation end products) to activate multiple signalling pathways (e.g., NF-κB, NJK, p21RAS) [48]. Another important source of free radicals in CKD is activation of RAAS and increased expression of xanthine oxidase (XO), which also increases uric acid production [6]. Furthermore, by stimulating various reductases (e.g., aldose reductase), carbonyl stress is increased (↑AGE). However, carbonylation of proteins is an irreversible process. Although oxidized proteins can be degraded, the ability of proteasomes to remove them is limited and depends on the degree of protein oxidation [46]. Therefore, oxidized proteins can disrupt the function of different organs, including impairing saliva secretion [20,21,49]. The prolonged accumulation of glycooxidation products (especially AGE) may also enhance the infiltration of macrophages and neutrophils in the parenchyma of the salivary glands, increasing, on a positive feedback, the production of free radicals [48]. In our study, the potential relationship between protein glycooxidation and salivary gland dysfunction in CKD children may be indicated by negative correlations between NWS/SWS flow and the fluorescence of oxidative modification products. Importantly, no such dependence was observed in the control group.

However, it is not proteins but lipids that are particularly susceptible to oxidation. In our study we assessed the concentration of 4-HNE protein adducts and 8-isop, which was significantly higher in NWS and SWS of CKD children with reduced saliva secretion. It was shown that lipid peroxidation products modify the physical properties of cell membranes. This increases the cell membrane permeability and reduces the difference in electrical potentials on both sides of the lipid bi-layer. Lipid oxidation may also affect the secretory function of the salivary glands, particularly since oxidized lipids induce further oxidative damage to proteins and DNA [20,21,49]. In our study, salivary 8-isop and 4-HNE correlated negatively with salivary flow, protein content and α-amylase activity. Increased levels of malondialdehyde (MDA) were observed in salivary glands of rats with experimental chronic kidney disease [50] as well as in saliva of children with CKD [3]. 4-HNE protein adducts may also increase the expression of matrix metalloproteinases, which not only damage the parenchyma of the salivary glands but also the nerves involved in salivary secretion [21,51]. However, this issue requires further research, especially in the context of CKD.

Binding of various neurotransmitters to salivary gland duct/secretory acini receptors initiates the excretion of primary saliva. At the parasympathetic nerve endings, NO is secreted, which by increasing the level of calcium ions is responsible for opening water channels (aquaporins) [52]. It is therefore not surprising that the concentration of total NO was significantly lower in CKD children with hyposalivation. Indeed, in children with CKD there is a reduced NO synthesis and increased endothelial production of the endogenous NO synthase inhibitor (asymmetric dimethylarginine, ADMA) [53]. However, the decrease in NO bioavailability can be explained not only by the impairment of endothelial cells but also by increased production of peroxynitrite. It is formed in the reaction of nitric oxide with superoxide radical anion. The resulting peroxynitrite is a weaker vasodilatant (than NO), but also a much stronger and more stable oxidant. It has been shown that peroxynitrite oxidizes thiol groups of proteins, initiates lipid peroxidation and inhibits mitochondrial respiratory chain (also in the salivary glands [14,20,21]). In our study, peroxynitrite levels were significantly higher in NWS, SWS, and plasma of CKD children with hyposalivation compared to those with normal salivary secretion. Although our methodology is routinely used to assess nitrosative stress in frozen saliva samples [54,55], it should be remembered that NO and peroxynitrite have a very short half-life and this may understate the results obtained.

An important part of the study was also the evaluation of the saliva-blood correlation coefficients. In children with CKD, salivary glycooxidation products, lipid oxidation damage and nitrosative stress products did not correlate with their plasma content. Thus, salivary biomarkers do not reflect the central redox homeostasis of CKD children. It is well known that the products of protein and lipid oxidation are not only produced in the salivary glands. By passive and active transport, these compounds can be transported from plasma to the oral cavity. Nevertheless, our study indicates that oxidative/nitrosative stress are different at the local (NWS, SWS) and central (plasma) levels. The influence of several environmental factors on salivary redox homeostasis is not without significance [19]. The oxidative-reductive balance of the oral cavity may also be varied by a distinctive microbiota composition of CKD patients [56]. 

However, correlations between salivary and plasma oxidation products were observed in the control group. Indeed, in healthy children, adults and the elderly, the concentration of oxidative stress products in NWS generally reflects their plasma content [28]. However, also in some systemic diseases, salivary oxidation products correlates with their blood level [32,55]. Salivary redox biomarkers can therefore be used in the diagnosis of systemic diseases, but only when salivary hypofunction is not present [57].

Hyposalivation was also observed in adults with CKD [23,58,59]. Although the cause of disturbed salivary gland function in CKD is still unknown, it is assumed that apart from changes in NO bioavailability, pharmacotherapy may also affect salivary secretion. Indeed, numerous drugs, including those used in CKD therapy, may influence the quantitative and qualitative composition of saliva. Some of them affect the water-electrolyte balance of salivary glands, while others block muscarinic/adrenergic receptors involved in the initiation of saliva secretion. Since pharmacotherapy is one of the major causes of hyposalivation [16,27], in this study we excluded children taking 5 and more medications. What is important, we did not observe any significant differences in salivary flow/redox biomarkers depending on the number of drugs.

Finally, please note the limitations of our manuscript. Firstly, the method of saliva collection using citric acid may affect the pH of the stimulated saliva. For analysis of components in SWS, mastication of no-taste gum could be a better method. Secondly, we only assessed the selected biomarkers of oxidative/nitrosative damage. Therefore, we cannot fully characterize salivary redox homeostasis in CKD children. Since oxidative stress promotes inflammation, the assessment of pro-inflammatory mediators in saliva is also indicated. Moreover, we cannot eliminate the impact of pharmacotherapy on saliva secretion and composition. Nevertheless, the study was carried out on children from whom non-stimulated and stimulated saliva as well as plasma were taken. The study and control groups are also carefully selected for accompanying diseases and periodontal status.

## 5. Conclusions

Chronic kidney disease is associated with salivary gland dysfunction and increased oxidative and nitrosative damage. Oxidation of salivary proteins and lipids increases with the progression of the disease and the degree of salivary gland damage. The assessment of salivary gland function should be an integral part of a dental examination in patients with CKD. Antioxidant supplementation may be considered in CKD children; nevertheless, further research is necessary, especially in a larger population of patients.

## Figures and Tables

**Table 1:** Clinical characteristics of children with chronic kidney disease (CKD) and healthy controls.

		C (*n* = 30)	CKD NS (*n* = 18)	CKD HS (*n* = 12)	ANOVA *p*
NWS flow (mL min^−1^)	mean ± SD	0.495 ± 0.1	0.338 ± 0.09	0.138 ± 0.04	<0.0001
	min	0.292	0.219	0.0730	
	max	0.682	0.494	0.199	
Men *n*		15	7	8	NA
Age (years)		13 ± 3.5	14 ± 3.2	12 ± 3.7	NS
CKD *n*	stage 1	-	6	0	NA
	stage 2	-	5	0	NA
	stage 3	-	7	0	NA
	stage 4	-	0	6	NA
	stage 5	-	0	6	NA
eGFR (mL/min/1.73 m^2^)		136 ± 6.9	84 ± 43	18 ± 7.4	< 0.0001
Serum creatinine (mg/dL)		0.41 ± 0.09	1.2 ± 0.54	4.6 ± 0.58	<0.0001
Serum urea (mg/dL)		18 ± 2.6	44 ± 4.7	124 ± 13	<0.0001
Albuminuria (mg/24 h)		8 ± 0.9	51 ± 23	815 ± 236	<0.0001
Proteinuria (mg/24 h)		58.4 ± 3.5	403 ± 165	845 ± 248	<0.0001
Hgb (g/dL)		14.5 ± 0.3	13 ± 0.49	11 ± 0.51	<0.0001
Hct (%)		39.7 ± 1.1	38 ± 1.2	33 ± 1.3	<0.0001
Serum iron (μg/dL)		82 ± 2.1	68 ± 6.7	91 ± 8.6	<0.0001
Hypertension *n*		-	1	11	NA
Dialysis *n*		-	0	6	NA
Drugs per day *n*	0	-	2	0	NA
	1–2	-	10	4	NA
	3–4	-	6	8	NA
Drugs *n*	iron	-	9	10	NA
	loop diuretics	-	10	9	NA
	ACEI	-	10	9	NA
	β-blockers	-	3	5	NA
	CCB	-	3	3	NA

ACEI—Angiotensin-converting enzyme inhibitors; C—Healthy controls; CCB—Calcium channel blockers; CKD NS—CKD patients with normal salivary secretion; CKD HS—CKD patients with reduced salivary secretion; NA—not applicable; NWS—Non-stimulated whole saliva; eGFR—estimated glomerular filtration rate; Hct—Hematocrit; Hgb—Hemoglobin.

**Table 2:** Dental examination of children with chronic kidney disease (CKD) and healthy controls.

	C (*n* = 30)	CKD NS (*n* = 18)	CKD HS (*n* = 12)	ANOVA *p*
DMFT	2.5 ± 0.5	2.7 ± 0.7	2.8 ± 0.6	NS
dmft	9.8 ± 0.5	10.1 ± 0.5	10.3 ± 0.7	NS
GI	0 ± 0.1	0 ± 0.2	0 ± 0.2	NS
SBI	0 ± 0.1	0 ± 0.1	0 ± 0.1	NS

C—healthy controls; CKD NS—CKD patients with normal salivary secretion; CKD HS—CKD patients with hyposalivation; DMFT—decay, missing, filled teeth (for permanent teeth); dmft—decay, missing, filled teeth (for milk teeth); NS—not significant; SBI—Sulcus Bleeding Index; GI—Gingival Index.

**Table 3:** Correlations between analyzed redox biomarkers and renal function of children with chronic kidney disease (CKD) and healthy controls.

	C (*n* = 30)			CKD (*n* = 30)		
	eGFR	serum Cr	serum urea	eGFR	serum Cr	serum urea
Dityrosine NWS	0.086	0.163	0.22	***−0.579***	***0.72***	***0.713***
Kynurenine NWS	0.17	0.246	0.057	***−0.562***	***0.71***	***0.691***
N-formylkynurenine NWS	0.225	0.322	0.118	***−0.527***	***0.641***	***0.585***
Tryptophan NWS	0.095	0.218	0.216	0.203	−0.102	−0.097
AGE NWS	0.228	0.041	−0.015	***−0.531***	***0.555***	***0.568***
Total thiols NWS	0.13	−0.164	−0.144	***0.431***	***−0.675***	***−0.649***
4-HNE NWS	0.135	0.154	0.163	***−0.512***	***0.533***	***0.476***
8-isop NWS	−0.017	0.096	−0.032	***−0.592***	***0.819***	***0.76***
NO NWS	−0.072	−0.089	−0.132	***0.609***	***−0.594***	***−0.531***
S-nitrosothiols NWS	−0.014	0.268	***0.543***	***−0.675***	***0.551***	***0.525***
Peroxynitrite NWS	0.039	−0.073	−0.236	***−0.537***	***0.631***	***0.579***
Nitrotyrosine NWS	0.194	0.018	−0.034	***−0.565***	***0.625***	***0.593***
Dityrosine SWS	0.037	0.076	−0.349	***−0.632***	***0.721***	***0.601***
Kynurenine SWS	0.217	−0.249	−0.146	***−0.48***	***0.586***	***0.626***
N-formylkynurenine SWS	0.219	***−0.456***	−0.028	***−0.457***	***0.509***	***0.49***
Tryptophan SWS	0.041	0.247	0.049	0.255	***−0.296***	−0.193
AGE SWS	−0.138	−0.134	−0.123	***−0.413***	***0.372***	***0.39***
Total thiols SWS	0.106	0.379	0.007	***0.674***	***−0.736***	***−0.657***
4-HNE SWS	−0.057	−0.251	−0.081	***−0.286***	***0.306***	***0.227***
8-isop SWS	−0.136	−0.306	0.203	***−0.417***	***0.464***	***0.55***
NO SWS	−0.123	0.176	***0.353***	***0.661***	***−0.52***	***−0.452***
S-nitrosothiols SWS	−0.484	0.106	0.257	***−0.357***	***0.448***	***0.417***
Peroxynitrite SWS	0.198	***−0.459***	−0.031	−0.29	***0.341***	***0.243***
Nitrotyrosine SWS	−0.068	−0.015	−0.156	***−0.41***	0.367	0.2

4-HNE – 4-hydroxynoneal protein adducts; 8-isop—8-isoprostanes; AGE—Advanced glycation end products; C—Healthy controls; Cr—Creatinine; CKD—patients with chronic kidney disease; eGFR—estimated glomerular filtration rate; NWS—Non-stimulated whole saliva; SWS—Stimulated whole saliva. Statistically significant correlations (*p* < 0.05) are highlighted as bold and italics.

**Table 4:** Correlations between analyzed redox biomarkers and salivary gland function and salivary pH of children with chronic kidney disease (CKD) and healthy controls.

	C (*n* = 30)				CKD (*n* = 30)			
	**NWS Flow**	**Total Protein NWS**	**SA NWS**	**pH NWS**	**NWS Flow**	**Total Protein NWS**	**SA NWS**	**pH NWS**
Dityrosine NWS	−0.092	−0.128	0.061	0.002	***−0.754***	***−0.633***	***−0.522***	***0.561***
Kynurenine NWS	−0.042	0.051	0.123	−0.152	***−0.722***	***−0.521***	***−0.362***	***0.388***
N-formylkynurenine NWS	−0.166	0.255	0.154	−0.133	***−0.663***	***−0.52***	−0.24	***0.545***
Tryptophan NWS	−0.009	−0.107	−0.073	−0.217	0.242	***0.363***	0.348	−0.14
AGE NWS	−0.111	−0.236	−0.063	−0.141	***−0.618***	***−0.358***	***−0.367***	***0.403***
Total thiols NWS	−0.013	0.265	0.063	−0.18	***0.6***	***0.395***	0.09	−0.34
4-HNE NWS	0.161	0.156	−0.12	−0.191	***−0.628***	***−0.632***	***−0.403***	***0.619***
8-isop NWS	0.163	0.335	0.043	−0.254	***−0.711***	***−0.385***	***−0.316***	***0.422***
NO NWS	−0.24	0.149	0.183	−0.093	***0.577***	***0.343***	***0.439***	0.032
S-nitrosothiols NWS	0.214	0.259	0.058	−0.332	***−0.725***	***−0.397***	−0.255	0.285
Peroxynitrite NWS	−0.069	−0.021	0.297	0.101	***−0.666***	***−0.872***	***−0.486***	***0.508***
Nitrotyrosine NWS	0.063	0.094	−0.282	0.538	***−0.594***	***−0.402***	***−0.354***	0.268
	**SWS Flow**	**Total Protein SWS**	**SA SWS**	**pH SWS**	**SWS Flow**	**Total Protein SWS**	**SA SWS**	**pH SWS**
Dityrosine SWS	−0.041	0.082	−0.118	0.152	***−0.534***	***−0.513***	***−0.584***	0.088
Kynurenine SWS	0.16	−0.168	0.155	0.039	***−0.466***	***−0.394***	***−0.473***	0.099
N-formylkynurenine SWS	0.24	0.06	−0.129	0.143	***−0.442***	***−0.482***	***−0.478***	−0.016
Tryptophan SWS	0.277	−0.301	−0.055	−0.192	0.026	0.083	−0.026	0.035
AGE SWS	***−0.488***	0.078	−0.051	−0.123	***−0.553***	***−0.401***	***−0.513***	−0.008
Total thiols SWS	0.037	0.177	−0.271	−0.202	***0.566***	***0.509***	***0.538***	−0.192
4-HNE SWS	−0.127	0.126	0.11	0.22	−0.119	0.02	−0.151	0.014
8-isop SWS	0.045	−0.114	−0.027	0.116	−0.216	−0.318	***−0.369***	−0.346
NO SWS	0.245	−0.267	−0.091	−0.066	***0.589***	***0.448***	***0.612***	−0.187
S-nitrosothiols SWS	0.152	0.302	−0.294	−0.051	***−0.466***	***−0.329***	***−0.47***	0.052
Peroxynitrite SWS	***0.349***	−0.001	0.036	0.239	−0.242	−0.297	***−0.516***	−0.095
Nitrotyrosine SWS	0.084	0.025	−0.003	0.074	−0.266	***−0.521***	−0.232	0.067

4-HNE—4-hydroxynoneal protein adducts; 8-isop—8-isoprostanes; AGE—Advanced glycation end products; C—Healthy controls; CKD—patients with chronic kidney disease; NWS—Non-stimulated whole saliva; SA—Salivary amylase; SWS—Stimulated whole saliva. Statistically significant correlations (*p* < 0.05) are highlighted as bold and italics.

**Table 5:** Correlations between salivary and plasma redox biomarkers in children with chronic kidney disease (CKD) and healthy controls.

	C (*n* = 30)		CKD (*n* = 30)	
	NWS & plasma	SWS & plasma	NWS & plasma	SWS & plasma
Dityrosine	***0.825***	−0.106	0.173	0.106
Kynurenine	***0.698***	0.140	***0.504***	***0.629***
N-formylkynurenine	0.327	−0.019	0.268	0.060
Tryptophan	***0.507***	0.208	***−0.374***	0.043
AGE	***0.781***	***0.461***	−0.268	0.314
Total thiols	−0.042	0.084	***0.387***	0.335
4-HNE	***0.473***	0.210	0.257	0.262
8-isop	0.041	−0.208	0.349	0.350
NO	0.030	−0.209	0.016	−0.078
S-nitrosothiols	−0.045	0.174	−0.059	0.025
Peroxynitrite	0.121	−0.340	0.224	0.334
Nitrotyrosine	0.096	−0.054	0.165	0.140

4-HNE—4-hydroxynoneal protein adducts; 8-isop—8-isoprostanes; AGE—Advanced glycation end products; C—Healthy controls; CKD—Patients with chronic kidney disease; NWS—Non-stimulated whole saliva; SA—Salivary amylase; SWS—Stimulated whole saliva. Statistically significant correlations (*p* < 0.05) are highlighted as bold and italics.
